# Modeling the Steady-State Effects of Mean Arterial Pressure on the Kidneys

**DOI:** 10.1109/OJEMB.2020.3036547

**Published:** 2020-11-06

**Authors:** Benjamin J. Czerwin, Sandip Patel, Caitlyn M. Chiofolo, Jiayao Yuan, Nicolas W. Chbat

**Affiliations:** Columbia University 5798 New York NY 10027 USA; Quadrus Medical Technologies New York NY 10001 USA; Quadrus Medical Technologies New York NY 10001 USA; Columbia University 5798 New York NY 10027 USA

**Keywords:** Glomerulus, human, kidney, model, pressure, GFR, MAP

## Abstract

*Goal:* We describe the relationship between mean arterial pressure (MAP) and glomerular filtration rate (GFR) since therapies affecting MAP can have large effects on kidney function. *Methods:* We developed a closed-loop, steady-state mechanistic model of the human kidney with a reduced parameter set estimated from measurements. *Results:* The model was first validated against literature models. Further, GFR was validated against intensive care patient data (root mean squared error (RMSE) 13.5 mL/min) and against hypertensive patients receiving sodium nitroprusside (SNP) (RMSE less than 5 mL/min). A sensitivity analysis of the model reinforced the fact that vascular resistance is inversely related to GFR and showed that changes to either vascular resistance or renal autoregulation cause a significant change in sodium concentration in the descending limb of Henle. *Conclusions:* This model can be used to determine the impact of MAP on GFR and overall kidney health. The modeling framework lends itself to personalization of the model to a specific human.

## I. Introduction

Organ diseases altering blood pressure directly affect kidney function, since the renal artery is the blood supplier of the kidneys. The kidneys are a vital organ, whose main functions are to regulate water and electrolyte balance, and to excrete metabolic waste and bioactive substances [1]. Determining an appropriate mean arterial pressure (MAP) for renal perfusion and glomerular filtration rate (GFR) is significant for a doctor administering therapy [2].

There are several steady-state human models of the kidney that have been developed based on adjustments of rat models [3]–[5]. These models, using partial differential and algebraic constraint equations, adapt rat vessel dimensions to those of humans and then adjust certain transporter density parameters to match desired human outputs. The complex transport phenomena that characterize the kidneys are described by many parameters (coefficients) in transport equations. Models with a high number of parameters are prone to overfitting. Moss *et al.* [6] use a lumped parameter approach to dynamically model an entire rat kidney that reduces the number of parameters, but it is naturally not suitable for human applications. Hallow *et al.* [5] modeled hyperabsorption in human diabetic kidneys, focusing only on water and sodium transport. This model assumes negligible pressure drop across the glomerulus, limiting the modeling of glomerular filtration. Another model from Hallow and Gebremichael [7], describes the development of what they deem a *core model*, one that can be used as a starting point for different studies and modeling endeavors. Their model is similar to [5], but may suffer from possible overfitting due to a large number of parameters, 20 of which were simply tuned to make the data fit their model. In this work, however, we have used a minimal number of parameters to capture the relationship between mean arterial pressure and GFR. Uttamsingh *et al.* [8] uses a piecewise linear function adapted from a dog to model GFR as MAP changes in humans. However, this approach is prohibitive in simulating impaired feedback and its effect on GFR. Other models, as also mentioned in [7], are phenomenological models, which suffer from the inability to determine which specific mechanistic segments of the kidneys have changed. The parameters of a physiological model, on the other hand, when estimated, indicate specific kidney insults, as diseases are represented by parameter alterations. Currently, the MDRD (Modification of Diet in Renal Disease) is the method most used in estimating GFR, as described in [9]. It is also described here however, that this method is fraught with several shortcomings, most notably it is very inaccurate when GFR is above 60 mL/min/1.73m^2^. Therefore, in normal kidney function, this equation is not reliable, and often labs simply report GFR above 60 mL/min/1.73m^2^ as normal. Further, this equation has no predictive capabilities since the inputs into this equation (age, sex, race, and creatinine) are not described functionally as related to blood pressure or other variables. Another commonly used equation for estimating GFR is CKD-EPI (Chronic Kidney Disease Epidemiology Collaboration). This equation is largely cited as being more accurate than the MDRD equation [10]. However, with identical inputs, it has similar drawbacks in predicting GFR as the MDRD equation.

We develop a human kidney model that uses a minimal set of adjustable parameters while still capturing the essential physiology and avoids overfitting. We use constrained optimization to determine the parameter values. Our steady-state, closed-loop (i.e., with feedback) model is a set of algebraic equations produced by lumping several spatial locations together, thus minimizing our equation set and parameters. We calculate a pressure at the glomerulus, removing the negligible pressure drop assumption across the glomerulus. In doing so, we are able to determine GFR while making use of the Starling equation [1]. The model is validated against real human data collected from ICU patients [11] and published studies [12]. This model can aid in renal therapy via generation of a GFR-MAP relation and simulation of impaired feedback and resistance change effects that mimic therapies. The model could further be personalized, using parameter estimation techniques, to determine the optimal therapy for an individual patient.

## II. Materials and Methods

We first describe some model assumptions, then the formulation of model equations, estimation of model parameters, and finally the physiological feedback mechanisms. The nonlinear algebraic system of equations for this model comprising ($1$)–(12) are based on kidney physiology and are derived via continuity equations. The equations are solved with Newton's method in MATLAB.

With the aim of assessing the relation between blood pressure and GFR, we model the movement of fluid throughout the kidney. The flow of fluid throughout the kidney can be altered by tubuloglomerular feedback (TGF), which is a function of sodium concentration in the distal tubule. Hence, since we model TGF, we also model the movement of sodium.

**Fig. 1.**
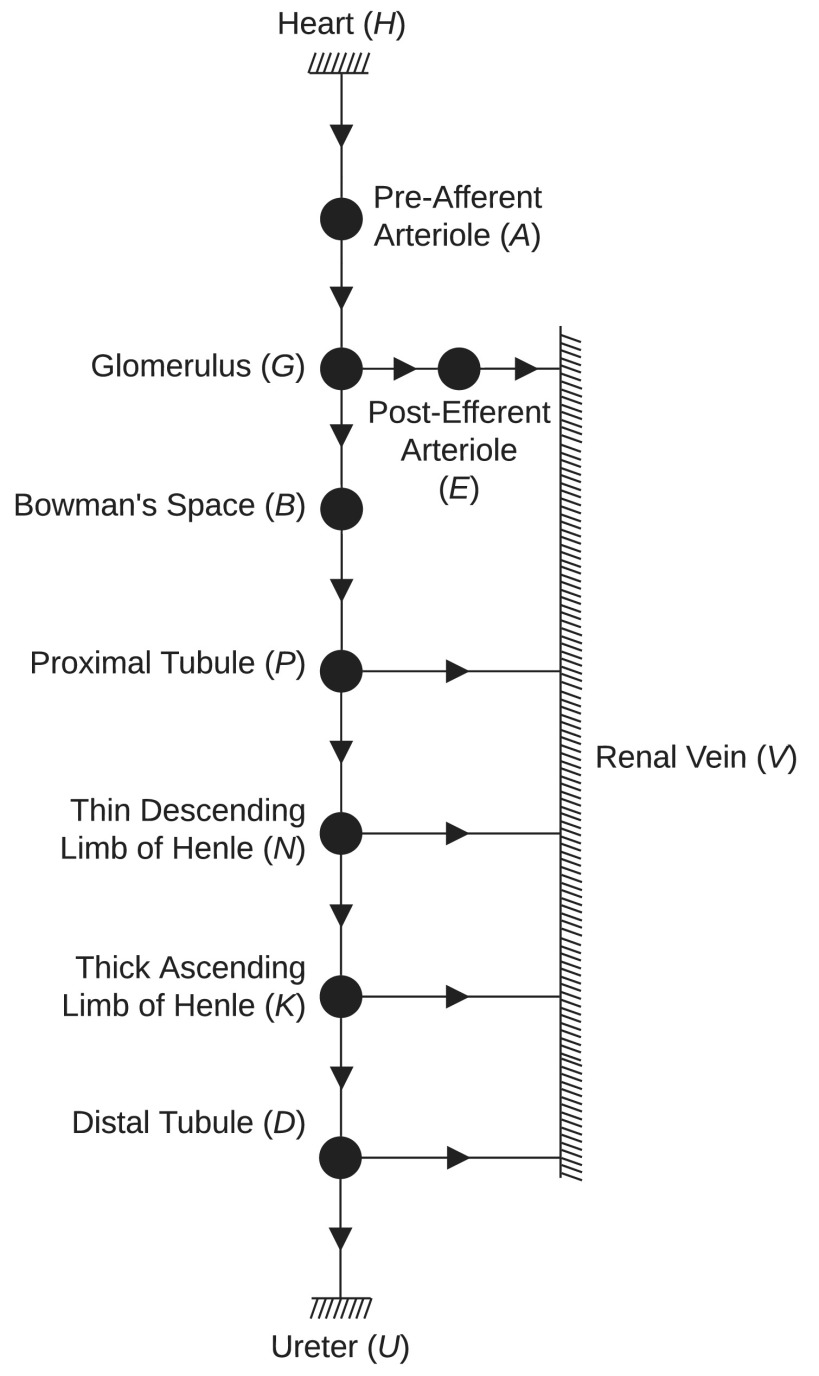
Kidney node schematic. Arrow directions indicate positive flow.

To accomplish this modeling task, the kidney is discretized into several spatial locations (nodes) based on physiological similarities between nodes, characterized by reabsorption paths. A schematic of the considered spatial nodes is shown in Fig. 1 with arrows leading to node $V$ representing the reabsorption paths. Each node is characterized by a hydraulic pressure and a sodium concentration. Fluid and sodium flows between nodes with positive flows indicated by the arrows in the figure. A single nephron is modeled in detail and variables are then multiplied by two million [13] to give an indication of the total renal plasma flow, GFR, and urine output. At each node, a mass conservation equation is developed, describing the steady-state physics of the hydraulic pressure and sodium concentration at that node.

For healthy simulation (baseline) we use the following typical model input values: a mean arterial pressure (MAP) of $90$ mmHg, a ureter pressure of $0$ mmHg, a venous pressure of $3$ mmHg, and a venous sodium concentration of $140$ mEq/L. We also assume that one tenth of the cardiac output (CO) enters each kidney, with one two-millionth of this flow entering each nephron. Renal plasma flow (RPF) is assumed to be $55$% of the blood flow entering the kidney and flowing into the pre-afferent arteriole node $A$ in Fig. 1 [1]. Finally, we ensure that the sum of total kidney urine flow and total kidney venous return must equal total kidney plasma flow.

### A. Hydraulic Modeling

Fluid moves axially (through the vessels) due to a hydraulic pressure gradient. This flow is determined by the hydraulic resistance parameter. The hydraulic resistance is a function of fluid material property and vessel thickness. The flow of water between arbitrary nodes $u$ and $w$ via hydraulic pressure gradient is modeled by

\begin{equation*}
Q_{uw}^h = \frac{{{\mathcal{P}_u} - {\mathcal{P}_w}}}{{R_{uw}^h}},\tag{1}
\end{equation*}

where $\mathcal{P}$ is hydraulic pressure at node $u$ or $w$, $R_{uw}^h$ is hydraulic resistance from node $u$ to $w$, and $Q_{uw}^h$ is hydraulic flow from $u$ to $w$.

Glomerular filtration modeling has consisted of either considering many capillaries in parallel or lumping the entire set of capillaries into one compartment and assuming that pressure and resistance drops across the glomerulus are negligible [14]. We calculate a pressure at the glomerulus, removing the negligible pressure drop assumption across the glomerulus. We assume negligible resistance due to the large number of capillaries in parallel (effectively reducing resistance to a negligible number). At the second and third nodes of Fig. 1, the glomerular filtration (between nodes $G$ and $B$) is modeled via the Starling equation as seen in (2) below. This equation is equivalent to hydraulic fluid flow (where the glomerular filtration coefficient is included in the resistance term $R_{GB}^h$), with an added term for the oncotic pressures, $\pi $. This oncotic pressure is due to proteins that are not filtered at the glomerulus and remain in the blood vessels and therefore we assume a reflection coefficient of unity for this oncotic pressure. The equation is

\begin{equation*}
Q_{GB}^g = \frac{{{\mathcal{P}_G} - {\mathcal{P}_B} - {\pi _{GB}}}}{{R_{GB}^h}}.\tag{2}
\end{equation*}

A typical value of the oncotic pressure between the glomerulus and Bowman's space is ${\pi _{GB}} = 30$ mmHg [13] is used.

In subsequent nodes, fluid is reabsorbed in the bloodstream along the nephron from the proximal tubule ($P$), thin descending limb of Henle ($N$), and the distal tubule ($D$) as shown in Fig. 1 by the arrows going into node $V$. We follow the assumption that the cellular walls of the thick ascending limb of Henle (node $K$) are impermeable to water and therefore no fluid is reabsorbed from $K$. We define a reabsorption fraction at each node where fluid is reabsorbed, as a percentage of the incoming axial flow. This transverse reabsorption fluid flow from an arbitrary node $w$ back to the veins, $V$, is therefore described by

\begin{equation*}
Q_{wV}^r = r_w^h \cdot Q_{uw}^h,\tag{3}
\end{equation*}

where $r_w^h$ is the fluid reabsorption fraction at node $w$, and $Q_{uw}^h$ is the axial flow (along the nephron) into node $w$ from node $u$. The reabsorption fractions of $P$ and $D$ are nearly constant, regardless of fluid flow, in healthy cases, as described by [8]. For node $P$, a constant value of $0.75$ is used for the reabsorption fraction due to the glomerulotubular balance, where reabsorption fractions of fluid and sodium are approximately equal and invariant to GFR. For $D$, a constant value of $0.95$ is used for the reabsorption fraction, which changes with antidiuretic hormone levels, but not with fluid flow, into $D$. A constant antidiuretic hormone level corresponding to a $0.95$ reabsorption fraction of water from node $D$ was used. [8]. For node $N$, the reabsorption fraction is an inverse function of fluid flow into $N$ given by [8] as

\begin{equation*}
r_N^h = 0.65 - 0.01{\rm{\ }}Q_{PN}^h.\tag{4}
\end{equation*}

### B. Sodium Modeling

As previously mentioned, we model sodium in the nephron in order to model TGF. Since sodium is freely filtered at the glomerulus, we assume that the sodium concentration at Bowman's space (node$\ B$) and at the renal veins (node $V$) are equal.

Axial flow of sodium along the tubule is due to advection, which is the movement of solutes by bulk fluid flow. Advective flow between nodes $u$ and $w$ is modeled via this equation,

\begin{equation*}
J_{uw}^a = Q_{uw}^h \cdot \mathcal{C}_u^{{\rm{Na}}}\tag{5}
\end{equation*}

where $\mathcal{C}_u^{{\rm{Na}}}$ is the sodium concentration at node $u$ and $Q_{uw}^h$ is the hydraulic flow between nodes $u$ and $w$.

Sodium reabsorption flow is modeled similarly to fluid reabsorption, in that we define reabsorption fractions for each node along the nephron. The transverse flow of sodium from an arbitrary node $w$ back to the veins, $V$, is therefore described by

\begin{equation*}
J_{wV}^r = r_w^{{\rm{Na}}} \cdot J_{uw}^a,\tag{6}
\end{equation*}

where $r_w^{{\rm{Na}}}$ is the sodium reabsorption fraction at node $w$. The cellular walls at the thin ascending limb of Henle (node $N$) are impermeable to sodium and therefore the reabsorption fraction there is zero. For node $P$, we again use $0.75$, as fluid and sodium are reabsorbed at almost one to one ratio. According to [8], the reabsorption fraction at the thick ascending limb of Henle (node $K$) is approximately invariant to the flow into node $K$ and approximately equal to $0.80$ and as such $r_D^{{\rm{Na}}} = 0.8$. At node $D$, the reabsorption fraction is determined by another renal feedback mechanism, the renin-angiotensin mechanism where the sodium reabsorption fraction is modulated by aldosterone blood levels, as outlined by Uttamsingh *et al.* [8]. This is given by an inverse sigmoidal function of sodium flow into $D$,

\begin{equation*}
r_D^{Na} = \frac{{0.2268}}{{1 + {e^{\left({7J_{KD}^a - 7.7} \right)}}}} + 0.7316.\tag{7}
\end{equation*}

### C. Parameter Estimation: Hydraulic Resistance

Axial fluid flows are characterized by hydraulic resistances. Hydraulic resistances of the axial flows are calculated in two ways. Method 1 uses the Poiseuille equation assuming laminar flow where the resistance between nodes $u$ and $w$, is given by

\begin{equation*}
R_{uw}^h = \frac{{8\eta \bar{l}}}{{\pi {{\bar{r}}^4}}},\tag{8}
\end{equation*}

where $\eta $ is fluid viscosity, and $\bar{l}$ and $\bar{r}$ are the average length and radii of nodes $u$ and $w$, respectively. Given the variability in human nephron dimensions, even between neighboring nephrons, calculating resistances via method 1 may not yield resistances that achieve physiologically accurate pressures throughout the nephron. For instance, small changes in radius will produce large changes in resistance, as the radius is raised to the fourth power in (8). By assuming healthy pressures, a GFR of $120$ mL/min [13], healthy reabsorption fractions as discussed above, and a resistance from $G$ to $B$ of $R_{GB}^h = {10^7}$ s/mL/mmHg, the hydraulic resistances can also be calculated directly from the continuity equations (conservation of mass), which defines method 2. The resistances via methods 1 and 2 ought to be identical, naturally. We then use optimization techniques (described below) to find feasible dimensions (lengths and radii) of the vessels. Subsequent resistances could then be calculated using (8), with dimensions closely matching those calculated by the system of continuity equations.

In method 2, all axial hydraulic resistances (including those along the tubule, excluding $R_{GB}^h$) were calculated using a set of physiologically reasonable pressures that are given in Table I. The afferent arteriole resistance ($R_{AG}^h$) is modulated by tubuloglomerular feedback as well as the ascending and descending myogenic feedback mechanisms, and hence $R_{AG}^h$ varies with pressure. The $R_{AG}^h$ calculated from the equations is after feedback modulation. The baseline value of $R_{AG}^h$, in the absence of feedback, was estimated from [15] to be $R_b^h = 2.78 \times {10^6}$ s/mL/mmHg.

**TABLE I:** Pressure Values Used in Resistance Calculation

**Node**	$\mathcal{P}$ **(mmHg)**	**Notes**
$A$	77	Estimated within feasible range
$G$	55	Given from [13]
$E$	17	Estimated from [1]
$B$	15	Given from [13]
$P$	10	Pressure from $P$ to $D$ decreases along tubule between $15$ mmHg at $B$ and $0$ mmHg at $U$
$N$	7	
$K$	6	
$D$	4	

Our goal is to determine feasible scaling factors of the lengths and radii that will be used to calculate resistances via method 1. These calculated resistances will then be used in simulation using baseline parameters, outputting the prescribed pressures. The iterative constrained optimization technique minimized the sum of the squares of the difference between resistances estimated from method 1 (which uses the scaling factors to calculate resistance) and method 2 (which are the true resistances) by estimating the scaling factors. Baseline dimensions were taken from Layton and Layton [3]. These scaling factors (outputs of the optimization) are nondimensional constants multiplying baseline lengths and radii, bound to a range between $0.7$ to $1.2$, with an initial value of $1$. This range represents varying nephron sizes in humans [16].

The optimization results in Table II show a feasible solution, with a possible local minimum. In this problem, we were more interested in feasibility (satisfying constraints) rather than optimality, for lengths and radii (and thus a global minimum) was not necessary to find. These optimized parameters (scaling factors) are then used to calculate resistances, assuming Poiseuille flow with (8), which will induce feasible pressures throughout the system at a healthy ${\rm{MAP}} = 90$ mmHg.

**TABLE II:** Parameter Estimation Results

**Node**	**Lengths**			**Radii**			**Branch**	**Poiseuille Resistance**	**Optimized Resistance**
	**Literature [3]**	**Optimized**	**Scaling**	**Literature [3]**	**Optimized**	**Scaling**			
$P$	$1.7$	$2.0024$	$1.178$	$0.0019$	$0.0013$	$0.707$	$BP$	$1.06 \times {10^6}$	$5.00 \times {10^6}$
$N$	$0.32$	$0.3838$	$1.199$	$0.0013$	$0.0009$	$0.702$	$PN$	$2.51 \times {10^6}$	$12.0 \times {10^6}$
$K$	$1$	$0.7$	$0.700$	$0.0013$	$0.0013$	$0.982$	$NK$	$3.77 \times {10^6}$	$6.15 \times {10^6}$
$D$	$2$	$2.3947$	$1.197$	$0.0010$	$0.0011$	$1.116$	$KD$	$14.0 \times {10^6}$	$12.3 \times {10^6}$

Lengths and radii from the literature and the optimization (cm). The scaling of the literature value is also presented and given such that the value from the literature multiplied by the scaling is the optimized value. Resistances assuming Poiseuille flow calculated from values in the literature [3] before and after optimization (s/mL/mmHg) are also shown.

The resulting resistances in Table II are, in some instances, much larger than those estimated from the original dimensions. However, since all lengths and radii are bounded between $0.7$ and $1.2$ times their original values, they still represent plausible resistances for a human. The average percent error between resistances calculated from continuity equations versus those calculated from optimization is $5.2 \times {10^{ - 6}}$% indicating a good fit between the optimized values. Using the resistances in Table II, we arrive at accurate pressures throughout the system that match the prescribed pressures in Table I.

In addition to finding the resistances via optimization, we needed to find the hydraulic resistance from the heart (node $H$) to the pre-afferent arteriole (node $A$) for simulations where MAP is varied, since we could no longer use a predefined renal plasma flow, because this flow varies with MAP. A pressure drop from the heart (node $H$) to node $A$ was set to $13$ mmHg in accordance with [17]. With this pressure drop and a baseline cardiac output of $5.5$ L/min at an MAP of $90$ mmHg, the resistance from heart to pre-afferent arterioles ($R_{HA}^h$) was calculated to be $2.58 \times {10^6}$ s/mL/mmHg by rearranging ($1$) to solve for the resistance.

### D. Feedback Mechanisms

**Fig. 2.**
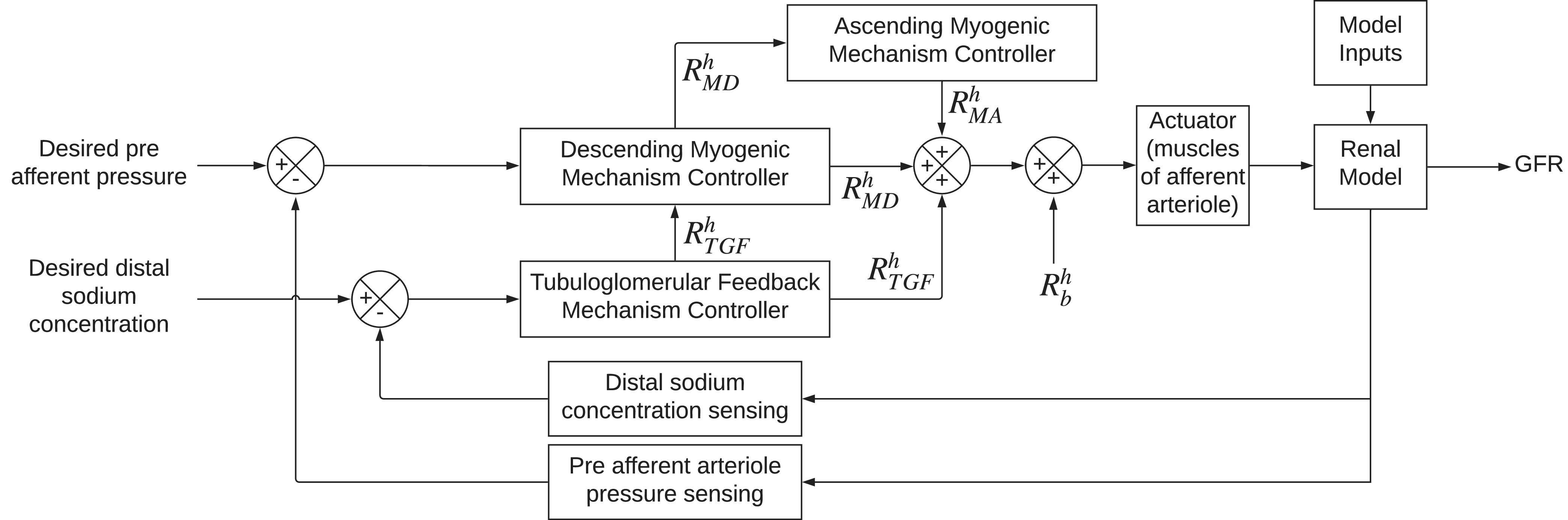
Feedback diagram for tubuloglomerular feedback and descending and ascending myogenic mechanisms. Model inputs include mean arterial pressure, ureter pressure, venous pressure and sodium concentration.

Since patient measurements typically occur at intervals longer than the transient response of the kidney, we are only interested in GFR after this transient response. As such, we adopt the steady-state feedback equations of [18], implemented by [19]. They included tubuloglomerular feedback (TGF) and descending and ascending myogenic feedback mechanisms. A feedback diagram is presented in Fig. 2. As shown, all feedback mechanisms share a common actuator that is the afferent arteriole muscles that change $R_{AG}^h$ by constricting or dilating. However, each controller has a different sensed input (located in the feedback paths in Fig. 2). TGF (10) senses $\mathcal{C}_D^{{\rm{Na}}}$ and imposes an additional resistance $R_{TGF}^h$ on the baseline resistance of $R_{AG}^h$, denoted by $R_b^h$, as $\mathcal{C}_D^{{\rm{Na}}}$ decreases. The descending myogenic mechanism (MD) senses pre-afferent arteriole pressure and imposes an additional resistance $R_{MD}^h$ when this pressure rises above $67$ mmHg, as seen in (11). Beyond $67$ mmHg, $R_{MD}^h$ continually increases, linearly with pre-afferent pressure, as the vessel continues to constrict. The ascending myogenic mechanism (MA) senses afferent arteriole resistances (determined by the descending myogenic mechanism and TGF, since MD is a function of TGF) and imposes yet another resistance $R_{MA}^h$, as shown in (12). The total resistance is hence given by the combined additive effect of all feedback mechanisms as

\begin{equation*}
R_{AG}^h = R_b^h + R_{TGF}^h + R_{MD}^h + R_{MA}^h\tag{9}
\end{equation*}

with,

\begin{align*}
&R_{TGF}^h = \frac{{0.1505}}{{1 + {e^{\left({4.8 - 40\mathcal{C}_D^{{\rm{Na}}}} \right)}}}}\tag{10}\\
&R_{MD}^h = 0.5 \cdot \left({R_b^h \!+\! R_{GE}^h \!+\! R_{TGF}^h} \right) \!\cdot\! \left({\frac{{{\mathcal{P}_A}}}{{67}} \!-\! 1} \!\!\right) \!\cdot H\left({{\mathcal{P}_A} \!-\! 67} \right)\tag{11}\\
&R_{MA}^h = 0.5 \cdot \frac{{R_b^h + R_{MD}^h}}{{R_{GE}^h}},\tag{12}
\end{align*}

where$\ R_{GE}^h\ $is the hydraulic resistance from glomerulus to post-efferent arteriole and $H({{\mathcal{P}_A} - 67})$ is the Heaviside function that is $0$ when ${\mathcal{P}_A}$ is below $67$ mmHg and $1$ otherwise. It can be seen that (12) is a function of $R_{MD}^h$ and subsequently $R_{TGF}^h$ as well. Equations (10) – (12) are multiplied by a feedback gain. When these gains are zeros, the system is open loop. For a healthy person, these gains are ones.

## III. Results and Discussion

### A. Validation

The model is validated against real patient data as well as other models in the literature. As will be detailed in the results below, we first compare our model outputs to the model outputs from [3] and [8]. We then validate against patient data by comparing model generated GFR vs. MAP to those of five intensive care unit patients with diagnoses unlikely to impact kidney function. Finally, we compare the same GFR vs. MAP curve to the data from 20 patients (across a large range of ages) reported in [12] over a two-hour study. None of these patients had clinical evidence of primary renal disease.

**Fig. 3.**
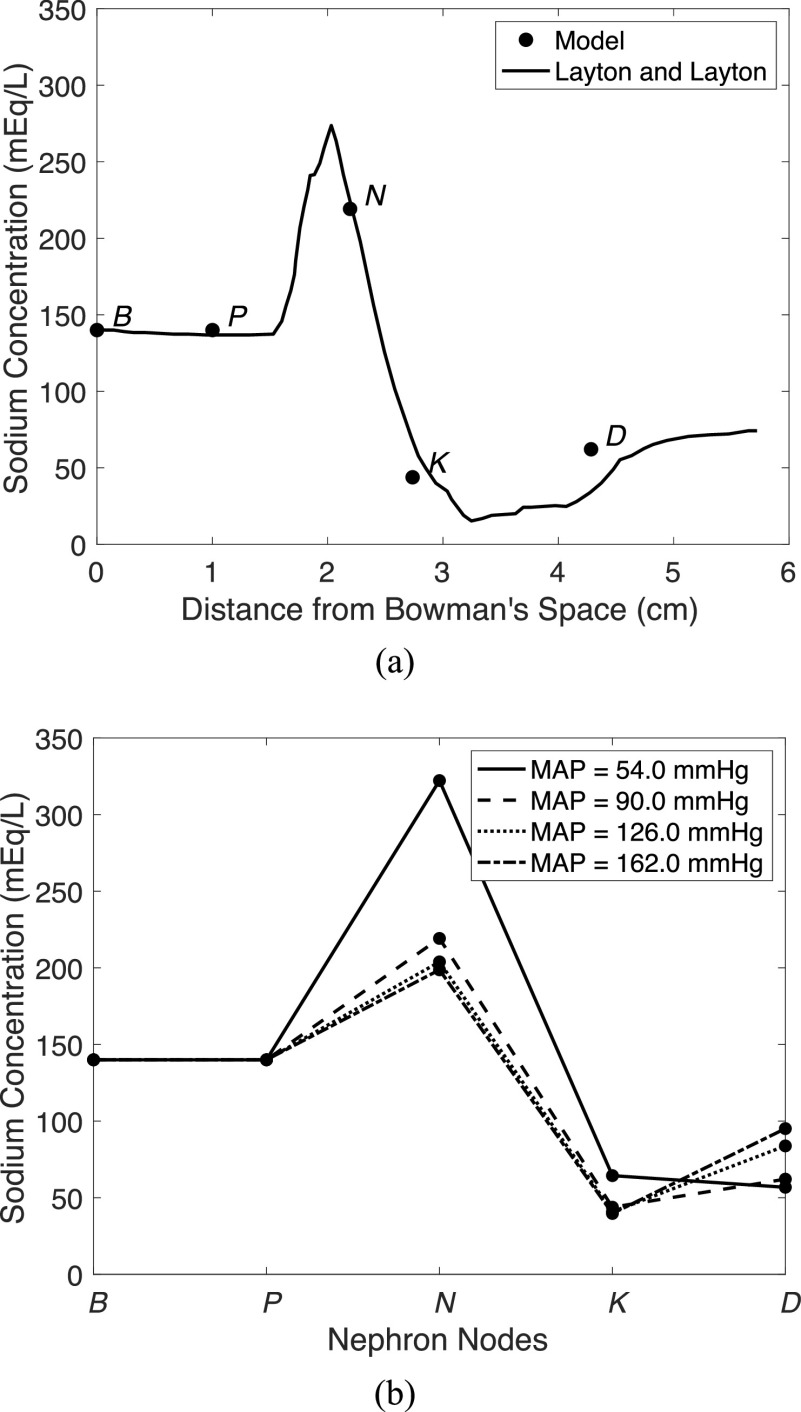
Model sodium concentration values along the tubule compared to [3] and over varied MAP inputs ranging from 54 to 180 mmHg.

We simulated the model under healthy conditions at ${\rm{MAP}} = 90$ mmHg (${\mathcal{P}_u}$ in ($1$) for the continuity equation at node $A$) for healthy cases and solve for all pressures, flows, and sodium concentrations at and between the nodes in Fig. 1. Sodium concentrations along the tubule were compared to Layton and Layton's continuous steady-state model [3] in Fig. 3a. As shown, the values are close in magnitude. This is a good indication that the concentrations of sodium in the tubule are physiologically sound. Of note, our model lumps the distal tubule and collecting duct into one node as also seen in [8], and therefore we see, in Fig. 3a, our sodium concentration at $D$ slightly differ from [3]. However, we observed little impact of the length of the $D$ node on the resulting sodium concentration and our model's determination of GFR.

Flow values of fluid and sodium between several nodes are compared to Uttamsingh *et al.* [8] in Table III, with percent differences reported. Our baseline (no parameter alterations) simulation results indicate good matching to those of Uttamsingh *et al.*, with an average percent difference of $7.7\% $. Our model explicitly includes resistances and pressures and uses the Starling equation, rather than the piecewise linear function used by Uttamsingh *et al.*, for calculation of GFR via (2), allowing us to simulate impaired feedback and observe subsequent changes in GFR, as presented in Section B *Analysis*.

**TABLE III:** 

**Branch**	**Uttamsingh *et al.* [8]**	**Model**	**% Difference**
$GB$	$125$	$115.47$	7.62
$PN$	$31.25$	$28.87$	7.62
$DU$	$1$	$0.850$	15.1
$PV$	$93.75$	$86.61$	7.62
$NV$	$10.55$	$10.43$	1.12
$DV$	$19.7$	$17.56$	10.9
Sodium Flows in mEq/min			
**Branch**	**Uttamsingh *et al.* [8]**	**Model**	**% Difference**
$PN$	$4.44$	$4.04$	8.97
$KD$	$0.89$	$0.50$	9.26
$PV$	$13.3$	$12.1$	8.83
$KV$	$3.55$	$3.23$	8.91
$DV$	$0.76$	$0.75$	0.52

After validating our model against other models, we then varied MAP over the range of $54$ to $180$ mmHg in increments of $10\% $ of the baseline ($90$ mmHg). The effect of MAP on sodium concentration along the nephron can be seen in Fig. 3b. With the rise in MAP, RPF also rises as given by ($1$). The increased RPF decreases fluid reabsorption from $N$ since the reabsorption fraction and fluid flow into node $N$ are inversely related, as described in ($4$). Therefore, $\mathcal{C}_N^{{\rm{Na}}}$ decreases as MAP increases, especially since sodium mass does not change in $N$ either, due to the impermeability of the tubule to sodium at this location.

At node $D$, sodium concentration increases with MAP. This is due to the inverse relationship between sodium reabsorption and sodium flow into $D$ as described in (7). This is expected, since TGF increases afferent arteriole resistance to decrease RPF and GFR when $\mathcal{C}_D^{{\rm{Na}}}$ is high.

**Fig. 4.**
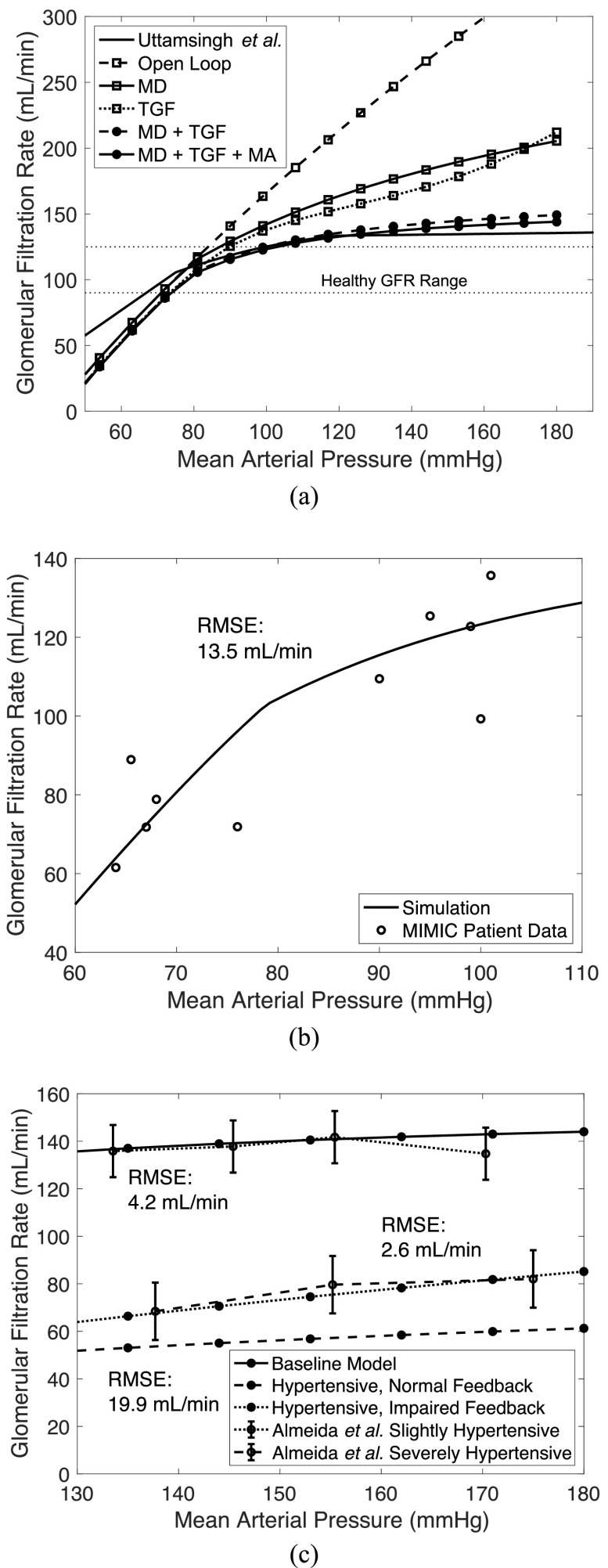
Glomerular filtration rate plotted as input MAP is varied: (a) under varying controllers active and compared to Uttamsingh *et al.* [8] simulation, (b) compared to Massachusetts’ ICU hospital patient data [11], and (c) in slightly and severely hypertensive cases as compared to real human data from Almeida *et al.* [12]. RMSE is root mean squared error. Descending myogenic mechanism (MD), ascending myogenic mechanism (MA), tubuloglomerular feedback (TGF).

GFR was also analyzed during a variation of MAP as shown in Fig. 4a. A spline interpolation was inserted between simulation points to generate a continuous curve. A healthy GFR range of $90$ to $120$ mL/min is highlighted between two horizontal dotted lines. Additional simulations are shown where different feedback mechanisms were disabled in Fig. 4a. As can be seen in the open loop system (dashed line with open square markers), after $85$ mmHg, GFR is already beyond the healthy limit of $120$ mL/min (now in the region of hyperfiltration) and climbs rapidly (almost linearly) as MAP increases. The descending (solid line with open square markers) and ascending myogenic mechanism are effectively inactive below ${\rm{MAP}} = 80$ mmHg and beyond this point, they begin to increase $R_{AG}^h$ continually as MAP increases. This increase in $R_{AG}^h$ decreases GFR for a given MAP. TGF (dotted line with open square markers) becomes less effective, saturating to a maximum $R_{TGF}^h$ value beyond which increased MAP values are no longer controlled by this mechanism, as shown by the line's change in slope with sufficiently large MAP. The ascending myogenic response is not simulated without the descending myogenic response and TGF, as $R_{MA}^h$ would be zero without the TGF. The descending myogenic response is simulated without TGF where $R_{TGF}^h = 0$, in (11). Uttamsingh *et al.*'s [8] equation (solid line with no markers) for GFR as MAP changes and our curve (solid line with markers, with all feedback mechanisms enabled) match very well between $80$ and $100$ mmHg, the healthy range of MAP. The ascending myogenic mechanism has the effect of decreasing GFR with increased MAP beyond $80$ mmHg, as demonstrated in [18]. As such, including the ascending myogenic mechanism causes the GFR vs. MAP curve to flatten or increase GFR much less for further MAP increases.

This model can also be used to study the well-known effect of systolic blood pressure on kidney function. Systolic blood pressure and pulse pressure (the difference between systolic and diastolic pressure) have been linked to arterial stiffness [20]. A change to systolic blood pressure can be simulated in the model via 1) an alteration of MAP (since MAP is a function of systolic pressure) directly affecting renal blood flow, and 2) an alteration of all resistances pertaining to arterioles. This model cannot differentiate between systolic and diastolic pressure explicitly, however, since it is a steady-state model.

**Fig. 5.**
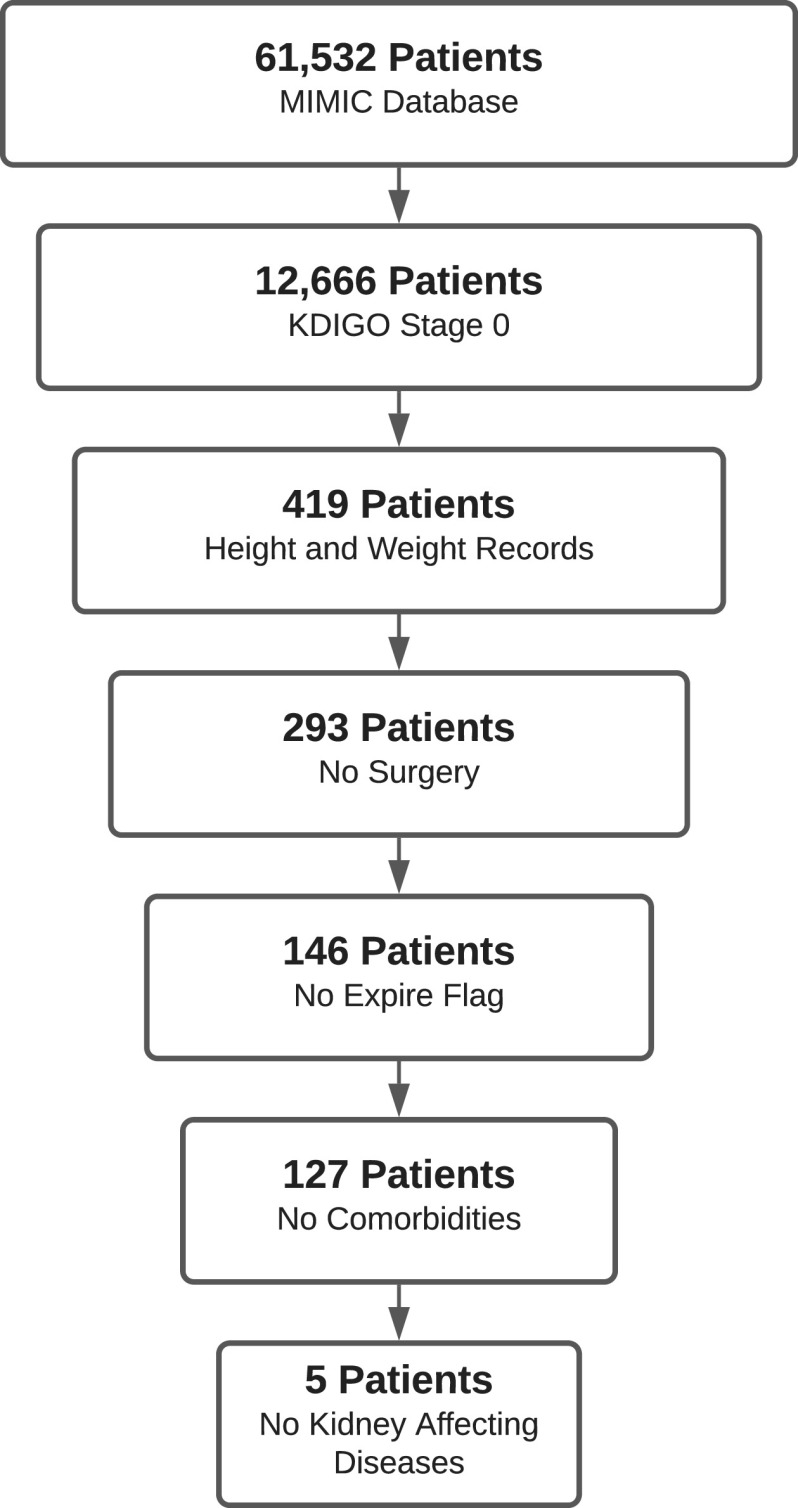
Patient inclusion criteria from MIMIC database. After removing patients with insufficient data for calculating a KDIGO (Kidney Disease Improving Global Outcomes) stage of 0, indicating no acute kidney injury, we had a set of 12,666 unique ICU stay IDs (identification number for each stay). Patients were further filtered from the database by retaining only those meeting the following criteria: patients who have height and weight records in order to remove GFR body surface area normalization in the data (419 remaining), patients who have not undergone surgery during a stay, which may affect GFR (293 remaining), patients who have not passed away during a stay (146 remaining), patients without diseases such as diabetes, cancer, pancreatitis, etc. (127 remaining), patients with diagnoses that would most likely not affect kidney function (5 remaining).

We next compared model-computed GFR to the GFR calculated using patient data from the MIMIC database [11] and utilizing the widely used Modification of Diet in Renal Disease (MDRD) formula [21] after removing the GFR normalization by patient body surface area. MIMIC was chosen since it is a large, diverse population database that is widely used and easily accessible. The database also includes multiple measurements from a single patient, at different instances, rather than aggregated or averaged population data only. All ICU stays are considered separately – even if the stay is a readmission of the same patient. Because we modeled a healthy individual, we want to validate on healthy patients. Since most patients in the MIMIC ICU data are critically ill, we sought to find relatively healthier patients, i.e., those with no acute or chronic kidney problems, and then filter out those with other kidney, heart, or severe illnesses that could impact kidney function. Patients were included according to the criteria described in the chart in Fig. 5.

Four of the patients remaining were male. The mean age was $34.2$, with standard deviation of $15.5$. The mean BMI (body mass index) was $21.4$, with standard deviation $4.9$. The diagnoses were altered mental status, diaphragmatic injury, and abnormal head CT. It is important to note that all patients were under the age of $60$, and patients, with the exception of one, were within a healthy BMI of 18.5 and 24.9. These two observations are important, as age can greatly affect GFR, generally diminishing with age, and a large BMI will result in a larger GFR than for a similar patient with a lower BMI since the MDRD equation, for calculating GFR, is normalized by body surface area, whereas our model outputs are not.

The associated blood pressure and GFR measurements were plotted for these ICU stay IDs against the simulated curve that can be seen in Fig. 4b. Several patients had more than one measurement of blood pressure and GFR, hence we see more than 5 data points. The data does not fit perfectly to our curve, as is expected without manual fitting or model fine-tuning, but it does show the general shape and magnitude of our curve. GFR can be altered by many factors that are not captured in the general model and therefore a more personalized model with parameters reflecting each patient would better fit the data for each patient. We observe an RMSE (root mean squared error) of all the data of $13.5$ mL/min. This is a reasonable number considering the data is from $5$ unique ICU stay IDs and there are healthy variations between patients in general due to natural variations in parameter values. We note also that the data fits the curve reasonably well in regions where feedback is not active (below ${\rm{MAP}} = 80$ mmHg) and areas where feedback is active (above ${\rm{MAP}} = 80$ mmHg).

The purpose of this validation against real patient data is to ensure that the model is able to capture the steady-state response of GFR to changes in MAP. With a low dataset number, we cannot expect to claim statistical conclusions on the model fit to patient data. However, we can claim the model is capable of describing this relationship within a reasonable range (15 mL/min GFR RMSE). Patient parameters can vary greatly from person to person as seen in [22], [23], and [24], describing the changes in nephron number, glomerular resistance, and renal autoregulation based on patient demographics alone. It is important to note that the validation data using healthy kidneys would correspond to normal parameters values, while diseased kidneys would correspond to altered parameters. As such, a more personalized patient-to-model fit requires parameter adjustment, even for healthy patients. One of the patients has a BMI outside of the healthy range and thus will have a GFR higher than the baseline, also contributing to an imperfect fit of the model to the data. As BMI increases, parameters in the model will change. This is due to the link between obesity and structural changes to the kidney that can cause decreased glomerular resistance and tubular flow in the loop of Henle [25], [26]. In the Analysis section we will observe how changing parameter values affects the model outputs. The current model can only describe certain clinical conditions. To address others, we would need to change several parameters to reflect healthy or diseased states of the patient. These parameters include aldosterone and antidiuretic hormone levels, glomerulotubular balance, reabsorption fractions, glomerular filtration resistances, and/or resistances. Several of these parameters, for instance, will change for diseases such as tubular necrosis or glomerulonephritis as described in [27].

We investigated the effect of administration of vasodilators in patients where there is a need to reduce MAP without negatively affecting GFR. We used real patient data from [12], where slightly and severely hypertensive patients were administered a stepwise infusion of sodium nitroprusside (SNP) to target acute blood pressure reductions. The slightly hypertensive patient group consisted of four men and six women with a mean age of 41.7±3.8 years (16-55 range), while the severely hypertensive patient group comprised four men and six women with a mean age of 38.5±4.3 years (15-58 range). We simulate SNP infusions as a change (reduction) in MAP. We plotted the collected data versus our model results in Fig. 4c. In the case of slight hypertension (dotted error bar line), our model (solid line), without any adjustment to vascular resistances or feedback gains, fits the data well with an RMSE of $4.2$ mL/min. To model severe hypertension, a sixfold increase of all vascular resistances ($R_{HA}^h$, $R_{AG}^h$, $R_{GE}^h$, and $R_{EV}^h$), corresponding to a decrease in vascular radii by $0.55$ times, was used. Almeida *et al.* [12] notes that the measurements of the severely hypertensive patients suggest that their feedback mechanisms are affected. Impaired feedback was then modeled by a decrease in feedback gains (multiplication by $0.5$) in all three feedback mechanisms described in (9)). After adjustments to vascular resistances and feedback gains, the model (dotted line) accurately represents the severely hypertensive patient GFR data (dashed error bar line) with an RMSE of $2.6$ mL/min, shown in Fig. 4c. In the case where vascular resistances are increased and feedback is not modulated (dashed line), the slope of the curve is nearly identical to the baseline curve, but the GFR values are too low to match the patient data having only an RMSE of $19.9$ mL/min. This is expected as the feedback mechanisms affect the slope of the GFR vs. MAP curve mostly. Feedback has a dampening effect on GFR as MAP changes, flattening the curve with increased feedback modulation as can be seen in Fig. 4a. This reinforces the hypothesis of Almeida *et al.* [12] that the feedback is impaired in severe hypertension, since here the model more closely matches severely hypertensive data after feedback modulation (Fig. 4c).

### B. Analysis

**Fig. 6.**
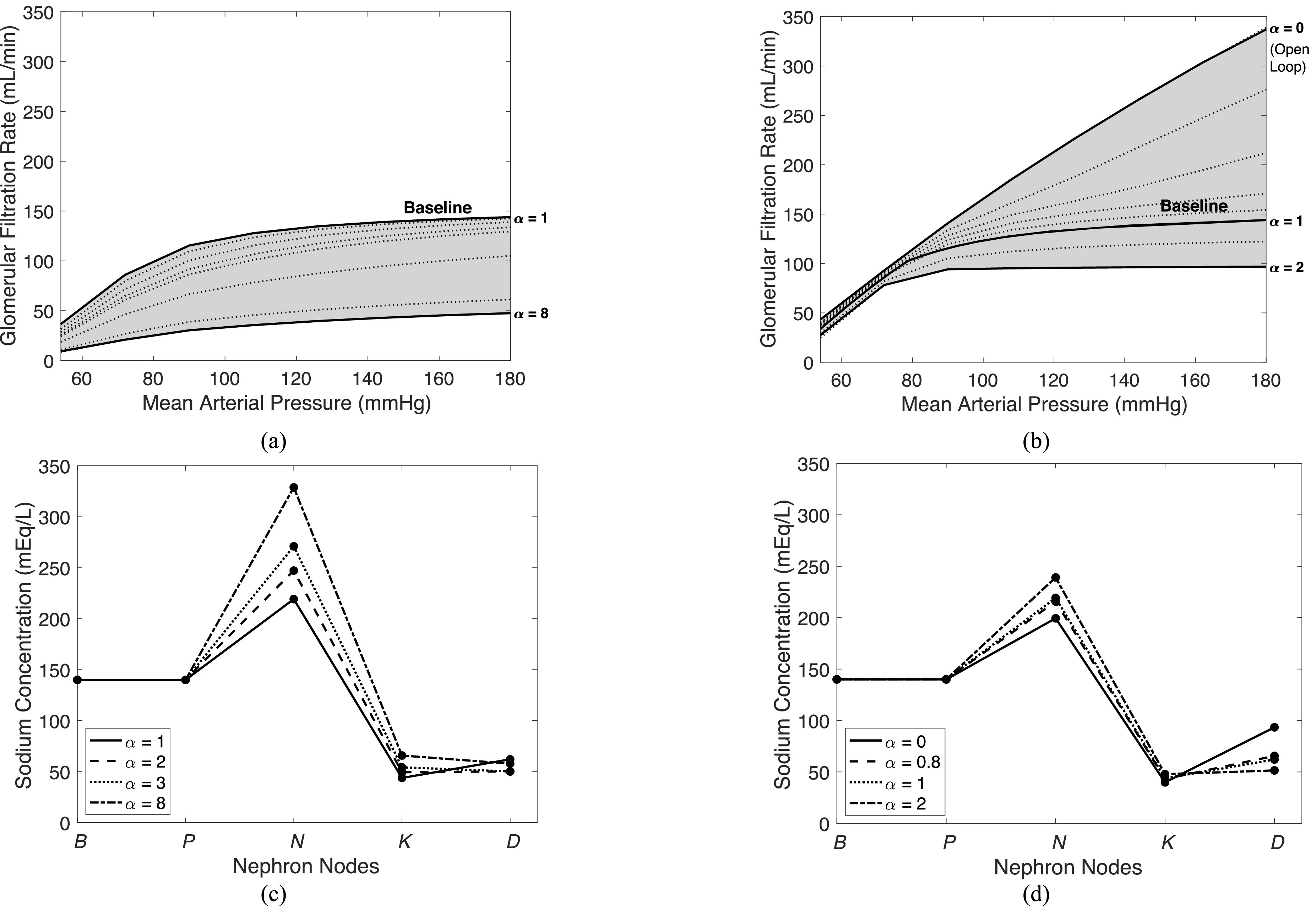
Sensitivity analysis results varying vascular resistance and feedback gains while monitoring GFR and sodium concentrations in the nephron. (a) GFR vs. MAP as vascular resistance is scaled by $\alpha $. (b) GFR vs. MAP as feedback responses are scaled by $\alpha $. (c) Sodium concentration along the nephron as vascular resistance is scaled by $\alpha $. (d) Sodium concentration along the nephron as feedback gains are scaled by $\alpha $.

We then conducted a sensitivity analysis to study how GFR and sodium concentration along the tubule are affected when vascular resistances and feedback gains are altered. Over a vascular resistance range of $1$ to $8$ times the baseline value, signifying worsening hypertension, we simulated the system over a range of MAP values. We also varied feedback gains over a range of $0$ to $2$ for the same MAP range, independently, simulating instances where feedback is altered, such as in hypertension. Results of varied vascular resistances and feedback gains are in Fig. 6, where $\alpha $ is a gain by which the vascular resistances or feedback gains were altered. For Fig. 6a and Fig. 6b, observing GFR, the dotted lines are individual simulations, and the solid lines delineate the region covered by the simulations.

We studied this increase in vascular resistance specifically for the case of hypertension [28], where vascular resistance is increased from the baseline value to $8$ times this value. The vascular resistances alter the curve such that GFR decreases with increasing vascular resistance as seen in Fig. 6a. A similar relationship was described by [29], where it was shown that increasing vascular resistances decrease the GFR. We see in Fig. 6b that feedback gains have a dramatic effect on the slope of GFR as MAP changes. This makes sense since impaired feedback will not effectively control high MAP values and thus GFR will rise more with MAP. In the open-loop system ($\alpha = 0$), GFR increases almost linearly with MAP as expected since $R_{AG}^h$ (modulated by the feedback) is severely decreased, as also seen in Fig. 4a. This reinforces the importance of feedback to maintain GFR during high MAP periods. Overall, GFR is more sensitive to impaired feedback as compared to changes in vascular resistances as seen by the larger changes in GFR from equivalent changes to vascular resistances or feedback gains in Fig. 6a and Fig. 6b.

We also studied how the sodium concentration, along the tubule, is affected by changes in vascular resistances and feedback gains, specifically at ${\rm{MAP}} = 90$ mmHg. These sodium values along the nephron are typically not measured for humans and therefore we can only provide insights. Results can be seen in Fig. 6c and Fig. 6d. The largest impacted sodium concentrations occur in the descending limb of Henle in response to changed vascular resistances and feedback gains. Sodium concentration in the distal tubule is also largely affected by feedback gain changes via TGF so it makes sense that sodium concentration would vary significantly in the same direction as this change.

As the vascular resistance increases, $\mathcal{C}_N^{{\rm{Na}}}$ (concentration of sodium in the $N$ node) increases as well. This is due to a decrease in fluid flow into $N$ and a subsequent increase in fluid reabsorption at $N$, as expected – see ($4$). This increased fluid reabsorption, in turn, increases the sodium concentration at node $N$. A similar, but less pronounced, effect can be seen when the feedback gains are increased due to the subsequent increase in $R_{AG}^h,$ as described by (9), which lowers the GFR and decreases reabsorption from node.

Given a decrease in feedback gains, $\mathcal{C}_D^{{\rm{Na}}}$ rises, due to the TGF response no longer effectively maintaining GFR. Impaired feedback decreases $R_{AG}^h$ and therefore fluid and sodium flow into $D$ increases. This subsequently decreases the reabsorption of sodium in $D$ and therefore raises $\mathcal{C}_D^{{\rm{Na}}}$.

## IV. Conclusion

We developed a closed-loop, nonlinear, steady-state, algebraic human kidney model with 30 parameters, several of which can be estimated using pressure values. Validation against other models in the literature and ICU patient GFR data, for critically ill patients without kidney injury, using normal kidney function parameters, has shown good matching with a 13.5 mL/min root mean squared error (RMSE). We further validated our model against a diseased case of hypertension where sodium nitroprusside was administered to reduce blood pressure. For mild hypertension, the baseline model accurately reproduced GFR as MAP changed. For severe hypertension, we increased vascular resistance and impaired feedback. The subsequent simulations showed good results compared to the data, RMSE of 4.2 and 2.6 mL/min for slightly and severely hypertensive patients respectively.

We investigated model response to changes in vascular resistances and feedback. We examined how GFR changed in response to these changes and noted that feedback had a dramatic effect on the slope of the GFR vs. MAP curve, due to the inability for the feedback to correct GFR with increasing MAP. As described in [29], we saw an inverse relationship between vascular resistance and GFR. We studied sodium concentrations along the tubule in response to these parameter changes because these values along the nephron are typically not measured for humans and therefore, the model can provide insights through simulation. We noted a significant change in sodium concentration in the descending limb of Henle, since fluid reabsorption is greatly affected by changes in GFR, shown in the sensitivity analysis to be largely affected by both vascular resistance and feedback.

In further studies, we will investigate the most common kidney diseases, glomerulonephritis and tubular necrosis, via parameter estimation techniques to generate personalized curves for individual patients. This would enable forecasting of variables in specific patients and/or what-if testing of therapy for specific patients.
